# Perceived Mental Workload and Psychological Variables in Elite Individual and Team Bulgarian Athletes: An Exploratory Study

**DOI:** 10.11621/pir.2025.0106

**Published:** 2025-03-01

**Authors:** Antonio Núñez, Milena Kuleva, Tatiana Iancheva, Alejandro García-Mas

**Affiliations:** a University of the Balearic Islands, Mallorca, Spain; b National Sports Academy “Vassil Levski”, So! a, Bulgaria

**Keywords:** mental workload, NASA-TLX, sports, anxiety, burnout

## Abstract

**Background:**

The concept of mental workload (MW) is a conceptual framework for evaluating the balance between the cognitive resources allocated to a task and those available to an individual. MW is influenced by the effort expended, the complexity of the task, and the individuals total processing capacity and resources.

**Objective:**

This paper aims to explore MW in the field of sport and its relationship with other psychological variables, such as anxiety and burnout. This approach is not widely used in this context so this analysis is considered novel and unique.

**Design:**

This paper is a cross-sectional study, with a sample selected by convenience sampling, consisting of Bulgarian athletes divided into three qualification categories: athletes competing at international competitions, athletes participating in national-level competitions, and non-professional athletes. The sample consisted of 107 athletes from various sports, with 62 male and 38 female participants. The instruments used to assess the different psychological variables included the NASA-TLX for MW and an abbreviated questionnaire derived from the SAS-2 for Anxiety and Burnout based on ABQ.

**Results:**

Athletes’ mental workload is multifaceted, influenced by physical and psychological factors, which may also act as a protective factor against anxiety. Team sports show higher levels of mental workload due to contextual uncertainty (entropy), which could reduce performance anxiety. Two athlete profiles emerged: a *bright side* (high effort, low anxiety) and a *dark side* (low effort, high anxiety).

**Conclusion:**

These findings contribute to existing knowledge in sports psychology, providing deeper insights into the role of mental workload in athletic preparation and its potential function as a protective factor against anxiety.

## Introduction

In the field of sports and physical activity sciences, it is common to measure and quantify physical workload and fatigue when, for example, planning and programming the different phases of the sports season (Freitas et al., 2016; Terrados et al., 2011).

The adaptation of physical load in sports is a common consideration in seeking to achieve optimal performance at the appropriate time of the season, for avoiding overtraining, sports injuries, andor burnout (Brenner & Watson, 2024; Glandorf et al., 2023). While this perspective focuses on physical aspects and it does not consider psychological aspects such as mental workload (MW). MW in sport is a neglected area of study either from a theoretical experimental standpoint. There are some existing studies related to the pre-fatigue induced by cognitive tasks done prior to to sporting tasks, observing increased mistakes in attempting free throws or passing a football (Alarcón et al., 2017; Alarcón et al., 2018). Additionally, there exists a few longitudinal assessments of MW linked with the physical load of rugby players (Barnard et al., 2020).

The origin of the conceptual framework for MW dates to the work of authors such as O’Donnell and Eggemeier (1986), who defined MW as the portion of a person’s limited capacity required at a given moment to perform a specific task. These authors described MW as a hypothetical construct representing the cost incurred by a human operator to achieve a particular level of performance. *Cost,* asdefined by these authors, is a subjectively perceived experience summarized by a variety of environmental factors related to demands of a given task.

Mental effort, in turn, refers to the volitional allocation of cognitive resources to meet the demands imposed by a task. The load, on the other hand, refers to the resources invested relative to those available and, therefore, depends on the effort exerted, the complexity of the taskand the total processing resources (Paas et al., 2003).

In short, MW is a dynamic construct that emerges from the interaction between task complexity and human resources, simultaneously reflecting and shaping these factors. Task complexity is determined by factors such as the task’s objectives, constraints on the number of items or elements, available time or time pressure, available space, the presence of distractors, and the availability of feedback (Cárdenas et al., 2015). Human resources refer to the individuals skills and abilities, learning history, motivation to complete the task, and subjective assessment of the task (Mischel & Shoda, 1995). It may be possible to manipulate MW in a manner similar to the management of physical load. Physical training load must provide sufficient stimulus to enable physiological adaptation but not so excessive as to impede that adaptation. Similarly, when managing mental workload, the stimulus inducing the load should fall within the same range. Having established this principle, perhaps the challenge may be reduced to (1) identifying which elements of the task determine its level of complexity or entropy and (2) individualizing the approach to generate an adequate stimulus (Cárdenas et al., 2015). Additionally, sports with higher levels of complexity or entropy may produce higher levels of WL.

There are activities that, despite lacking a prime motor component, can generate mental fatigue due to the significant mental resources required to carry out a given task. Additionally, as a complement to the previous definition, the total mental resources mobilized to address a task are also referred to as mental workload (MW).

Perceptions of MW haves been widely studied in different human performance tasks and fields including the study of automobile drivers and aviation pilots (Garcia-Mas et al., 2016; Jansen et al., 2016; Morgan & Hancock, 2011; Young et al., 2019). In the case of MW in the context of the workplace, MW has been related to burnout in some studies, studies that highlight that MW not only generates a short-term impact, but requires long-term adaptation and consideration with respect to labor policy. When these adaptations are compromised or fail due to poor planning, this may result in undesirable results, such as overtraining or burnout (Goodger et al., 2007).

According to Eastern European psychological literature, MW is primarily considered in relation to ergonomic standards of mental workload. Yakovlev (2002, 194 pp.) defined mental workload as “a random process of interaction (interpenetration) between external conditions (ecological, behavioral, situational), *i.e.*, objective workload demands, and internal conditions (functions, states, properties), *i.e.*, subjective manifestations that mobilize or drive the individual to a particular purposeful activity (activity)”.

In her online course, Ilieva (2023) defined MW as “the combination of all measurable influences on a person from the outside that affect them mentally”. Four main elements of mental workload were derived:

- Task requirements (sustained attention, responsibility, duration of activity, etc.).
- Physical conditions (lighting, microclimate, noise, odors, etc.).
- Social and organizational factors (organizational climate, teamwork, etc.)
- Social factors (outside the organization).
  Despite Yakovlev and Litovchenko (2007) indicating that the term *mental workload* has yet to receive official status in psychology, there are several *working* definitions of mental workload that suffice as a conceptual framework, which include:
- The recommendations of the Labor Research Institute have assessed the the combined effect of psychophysiological and hygienic factors on human performance and health. The primary indicator of the degree of MW on the organism is the pressure exerted on the emotional sphere, analytical functions, and attention (Kosilov, 1979).
- “A process of work which places such demands on the person that they are able to regulate the psychophysiological equilibrium of their organism only at a higher level or with the help of connecting functional systems” (Kunat, 1973, pp. 319-324).
- The result of situational and macro-temporal factors, such as individualpsychological characteristics, experience, degree of preparedness, along with factors that cause time-resistant changes in the level of mental stress, affects a person’s MW (Gorskaya, 1994).
- “Complexity, intensity, comprehensiveness, time constraints and semantics of the requirements imposed on the mental functions and processes of a person in the activity process” (Gazenko, 1987, 32 p.).

Four components of MW have been established the first of which includes the demands placed on the athlete’s personality and pattern of behavior by external and internal conditions. The second component is related to the evaluation and correction of actions, the level of activity of psychological functions, including a change in emotional excitement. The third component concerns the optimal level of emotional excitement, which is achieved by the presence of the athlete’s mental self-regulation skills. The fourth component involves mobilizing the athlete’s functional resources to achieve a set goal. If the first component represents a set of conditions that are currently independent of and uncontrolled by the athlete, the other three components represent processes controlled by the athlete.

MW is therefore a complex construct with a range of meanings that stem from a variety of external factors and conditions as well as the effects derived from the complexity of a given task, physical conditions, and social and organizational factors (Rogaleva et al., 2024). All of these factors coalesce to influence the behavioral patterns of an athlete including their perceived physical/mental effort required in the face of a given situation as well as the observable effects of fatigue, both mental and physical.

It is important to note that the MW does not occur in a vaccum as illustrated above. Moreover, it is reasonable to hypothesize that MW may relate to psychological variables, such as anxiety and burnout.

Competitive anxiety has been widely studied, and yet, from the Martens models (1977) through to those of Smith & Smoll (1990), it has become increasingly complex, leading to the identification of dimensions such as *Cognitive Anxiety*, *Somatic Anxiety*, *Deconcentration, while its* relationships with other variables, such as well-being and performance, have become more diffuse (Núñez & Garcia-Mas, 2017). Anxiety should not be considered a detrimental factor affecting performance or well-being in isolation, as this dynamic may be mediated by coping strategies or other psychological variables (Leguizamo et al., 2021).

Burnout has been studied both in the workplace and in sport due to its direct consequences on psychophysical well-being highlighting a causal relationship with overtraining (Glandorf, 2024), and other related factors such as sport injuries (Brenner & Watson, 2024; Gil-Caselles et al., 2024), often resulting in the abandonment of the activity as a consequence (Sors et al., 2020). Other research highlights the relationship between MW and burnout in the workplace and observes this inadequate adaptation of MW may occur due to either an excess or lack of demand volume both in terms of -quantitative and in terms of its complexity, qualitative output (Karwowski, 2006).

This research aimed to study MW exhibited by a sample of Bulgarian athletes from a variety of sports, and evaluate the levels of MW according sport, and the relationships between MW and the psychological variables anxiety and burnout.

We hypothesized that those types of sports that are relatively more complex requiring a greater number of real-time decisions, will obtain higher scores in MW. Regarding the relationships with the other variables, we speculated that anxiety and burnout be positively related to MW, and therefore, the greater the MW, the higher the levels of burnout and anxiety.

## Methods

### Participants

This paper’s design was a cross-sectional study, with a sample selected by convenience sampling. The athletes were recruited in Bulgaria through direct contact and on a voluntary basis. The participants in this study included student athletes from the National Sports Academy “Vassil Levski”, Sofia, Bulgaria, who represented competitive club teams and national teams.

The sample consisted of 107 athletes from a variety of sports, of which 62 were male and 38 female. The average age of the participants was 22.4, with an SD of 9.16, and an average number of years participating in their respective sport of 11.2 years, with an SD of 6.31. The inclusion criterion was regular physical activity. In this sense, a distinction was made between participants who regularly practiced physical activity at an amateur level (23.9%), national competitors (47.7%) and international competitors (28.4%).

The study was conducted individually with each participant, who provided written informed consent personally or, in the case of minors, through a parent or legal guardian, after being briefed about the study objectives. Data collection took place after training sessions or academic classes, following a specific sequence: participants first completed the NASA-TLX questionnaire, followed by anxiety and burnout assessment tools. All questionnaires were administered under research supervision, and proper instructions were provided. Subsequently, the collected data were coded and entered into a data processing system for analysis.

### Procedure

#### Questionnaires

An assessment protocol was developed based on the following three psychological variables with the incorporation of instruments established to measure each: *Mental Workload* (NASA-TLX), *Anxiety* (SAS-2), and *Burnout* (ABQ). Notably, since the primary objective of the study was to assess mental workload, the NASA-TLX measure was retained in its original length. Conversely, following the expert panel’s recommendations, the anxiety and burnout questionnaires were shortened by selecting items according to their factor loadings.

The Hart and Staveland, (1988) *NASA-Task Load Index* (NASA-TLX) instrument provided an overall workload score based on a weighted average of six dimensions: *Mental Demands*, *Physical Demands*, *Temporal Demands*, *Perceived Performance*, *Effort*, *and Frustration*. Each dimension is rated on a visual analog scale ranging from 0 to 100 points. The NASA-TLX questionnaire was translated into Bulgarian by three independent English-Bulgarian translators. The reliability analysis of the NASA-TLX yielded a Cronbach’s alpha coefficient of .704.

The application of the NASA-TLX requires two phases:

1. The first phase is called *pairwise weighting*. In this phase, the six dimensions of NASA-TLX are presented in pairs with the athlete selecting the more relevant dimension in each pair based on their prior experience and expectations. These selections are used to weigh the responses in the second phase.
2. The second phase involves completing the questionnaires with which the athlete rates each NASA-TLX dimension according to a visual analog scale (0–100 points).

The *Sport Anxiety Scale-2* (SAS-2, Smith et al., 2006) consists of 15 items, divided into three categories: *Somatic Anxiety*, *Worry*, and *Deconcentration*. Each of these items are rated on a 4-point Likert scale, ranging from 1 (Not at all) to 4 (Very much).

The *Athlete Burnout Questionnaire* (ABQ, Raedeke & Smith, 2001) consists of 15 items, divided into three dimensions: *Physical/Emotional Exhaustion*, *Reduced accomplishment*, and *sport devaluation*. Each item is rated using a 5-point Likert scale.

A shortened version of both scales was used due to factor load considerations. Based on the recommendations of an expert panel and item factor loadings, the two items with the highest factor loadings from each dimension of the construct were selected. This process resulted in reduced versions of both the SAS-2 and the ABQ, each comprising six items—two per dimension or factor.

## Results

The data were tested for normality using the Shapiro–Wilk test (Shapiro & Wilk, 1965). The NASA-TLX, SAS-2, and ABQ factors did not meet the normality assumption according to the Shapiro–Wilk (SW) test. Therefore, a non-parametric analysis was applied.

The Mann-Whitney test, Welch’s test, and Kruskal-Wallis test were used, along with Spearman correlations, to examine relationships and compare groups. Finally, a cluster analysis was conducted using K-means techniques. The statistical analysis was performed with Jamovi software (The Jamovi Project, 2023).

### Descriptive Statistics

The results indicated that international athletes were, on average, older and had more experience than the other groups *(Table 1)*. Descriptive statistics were not analyzed according to sport given the sample size for certain sports were too small, making separate analysis impractical.

**Table 1:** Mean values of the variables Age and Years of Sport experience for Qualification groups

Qualification	Age (Years)	Sport Experience (years)
International	23.0 (9.35)	12.5 (7.31)
National Competitor	22.4 (7.90)	11.5 (5.44)
Sports Activity	21.9 (10.9)	9.40 (6.25)

As shown in *Table 2*, the sports that, *a priori*, involve the highest physical demands are Canoe-Kayak, Wrestling, Skating, and Handball.

**Table 2:** Mean Values for NASA-TLX Factors on the pairwise weighting phase

Sport	Sample (n)	WL Global Index	Mental Weight	Physical Weight	Temporal Weight	Performance Weight	Effort Weight	Frustration Weight
Canoe-Kayak	1	88.3	3.00	5.00	1.00	2.00	4.00	.00
Ski board & Snow-	1	63.0	4.00	.00	1.00	2.00	3.00	5.00
Karate	11	71.7	2.00	3.10	1.10	2.80	3.60	2.40
Rhythmic Gymnastics	5	89.4	2.20	1.40	3.40	3.20	3.80	1.00
Volleyball	20	75.8	2.00	3.16	1.95	3.47	3.47	.94
Wrestling	1	96.3	2.00	5.00	1.00	4.00	3.00	.00
Basketball	18	76.8	1.44	3.56	2.56	3.44	3.50	.50
Handball	1	91.0	2.00	5.00	2.00	3.00	3.00	.00
Football	31	72.9	1.94	3.10	2.16	2.77	3.10	1.94
Dance	1	7.7	1.00	3.00	5.00	.00	3.00	3.00
Track athletics & Field	2	58.5	3.00	3.50	3.50	1.00	1.50	2.50
Taekwondo	1	88.0	1.00	4.00	3.00	3.00	3.00	1.00
Box	1	73.7	1.00	2.00	4.00	2.00	5.00	.00
Bowling	1	6.0	.00	3.00	1.00	5.00	4.00	2.00
Figure Skating	1	75.0	2.00	5.00	.00	3.00	3.00	2.00
Sambo	13	55.5	.77	3.15	2.15	3.62	3.31	2.00

The sports that require the highest mental demands are skiing and snowboarding. However, in general, the *Mental Demand factor* remained low across all sports in the *a priori* assessment.

The sport with the highest time pressure was dance, attributed to the need for precise synchronization with music to ensure optimal performance.

The sport that requires the most effort to achieve results was boxing.

Analyzing the Global Workload Index (Global WL Index), wrestling had the highest overall score, followed by handball. In contrast, sambo and track and field athletics were the sports with the lowest Global WL Index scores.

When analyzing the different weighted factors (using both the first phase and the second phase) in the NASA-TLX, we observed that the highest value was assigned to the importance of Performance, while the lowest value was assigned to the importance of Frustration *(Table 3).*

**Table 3:** Descriptives of the NASA-TLX (weighted) and SAS-2, ABQ scale factors

	Mean	SD	Min.	Max.
Mental Demands	119.67	111.736	0	500
Physical Demands	25.70	118.173	0	500
Temporal Demands	175.61	131.375	0	500
Performance	258.50	133.865	0	500
Effort	239.21	112.789	45	500
Frustration	48.50	79.495	0	475
Somatic Anxiety	2.29	.753	1.00	4.00
Worry	2.35	.875	1.00	4.00
Deconcentration	1.51	.651	1.00	4.00
Exhaustion	2.32	1.036	1.00	5.00
Devaluation of Sport	1.75	.808	1.00	4.50
Low Fulfillment	3.34	.657	2.00	5.00

The correlation matrix *(Table 4)* of the NASA-TLX and SAS-2 factors confirmed a negative correlation between Anxiety and Effort. Additionally, a positive correlation was observed between the Somatic and Worry factors in relation to the Frustration dimension of NASA-TLX.

**Table 4:** Correlations between NASA-TLX factors and SAS-2 factors means

	Men	Phys	Temp	Perf	Eff	Frus	Som	Wo	Dec
Men	—								
Phys	–.019	—							
Temp	–.178	.020	—						
Perf	–.019	–.120	–.349***	—					
Eff	–.014	.079	–.309**	–.005	—				
Frus	–.028	–.382***	–.062	–.273**	–.266**	—			
Som	–.048	–.072	.114	–.095	–.278**	.248**	—		
Wo	–.181	–.019	.061	–.119	–.293**	.224*	.445***	—	
Dec	–.024	–.123	.094	–.054	–.426***	.144	.271**	.331***	—

Note. * p < .05, ** p < .01, *** p < .001

Another correlation matrix was obtained when we analyzed the relationships between the ABQ factors *(Table 5)*. A negative correlation was observed between the Devaluation of Sports Practice factor and Mental Demand (NASA-TLX).

**Table 5:** Correlations between NASA-TLX factors and ABQ factors means

	Men	Phys	Temp	Perf	Eff	Frust	Exh	Dev	Lpf
Mental Weighted	—								
Physical Weighted	–.019	—							
Temporal Weighted	–.178	.020		—					
Performance Weighted	–.019	–.120		–.349***	—				
Effort Weighted	–.014	.079		–.309**	–.005	—			
Frustration Weighted	–.028	–.382***		–.062	–.273**	–.266**	—		
Exhaustion Mean	.160	.189	.135	–.026	–.033	–.081	—		
Devaluation Mean of Sports	–.195*	–.021	–.069	–.020	.009	.103	.121	—	
LPF	–.001	.058	.147	–.117	.273**	–.203*	–.128	–.266**	—

Note. * p < .05, ** p < .01, *** p < .001

The Low Commitment factor (LFP) correlated positively with Effort. It is important to note that the two selected items comprising this factor assess (1) goal achievement through sport. Therefore, a higher score corresponds to a higher perceived Effort and (2) suboptimal performance, where the item score is inverted. Therefore, the higher the perceived Performance, the higher the perceived Effort.

To analyze the overall correlations between the three assessment tools, we present another correlation matrix *(Table 6)*. It can be observed that a high Perceived Mental Load (global index) tended to decrease scores representing Worry and Distraction (SAS-2), while exhibiting a tendency to increase Exhaustion and Low Personal Fulfillment (ABQ).

**Table 6:** Correlation between NASA-TLX global index and SAS-2, ABQ factors

	WL Global	Som	Wo	Dec	Exh	Lpf	Dev
WL Global	—						
Som	–.131	—					
Wo	–.249**	.445 ***	—				
Dec	–.237*	.271**	.331***	—			
Exh	.250**	.138	.027	.081	—		
Lpf	.204*	–.097	–.158	–.152	–.128	—	
Dev	–.183	.006	.013	–.005	.121	–.266**	—

Note. * p < .05, ** p < .01, *** p < .001

### Group Comparison

Athletes grouped into team sports and individual sports were compared using the NASA-TLX and SAS-2 questionnaires. Levene’s test indicated a violation of the assumption of equality of variances (p < .05); therefore, Welch’s t-test was applied to the NASA-TLX scores. In contrast, for the SAS-2, Levene’s test did not indicate a violation of the assumption of equal variances (p > .05); consequently, the Mann-Whitney test was used.

Significant differences were observed between team and individual sports for the Global Mental Workload Index *(Table 7)*.

**Table 7:** Welch’s test for independent samples (team/individual) for NASA TLX factors

		Statistical	df	p
Mental Weighted	T de Welch	–.667	81.1	.506
Physical Weighted	T de Welch	–1.584	57.9	.119
Temporal Weighted	T de Welch	–1.386	76.3	.170
Performance Weighted	T de Welch	–1.605	71.7	.113
Effort Weighted	T de Welch	1.399	59.8	.167
Frustration Weighted	T de Welch	.607	59.3	.546
WL Global Index	T de Welch	–2.155	54.6	.036

*Note. H*_a_ *μ _individual_ ≠ μ _team_*

By identifying differences between groups in the Global Workload (WL) Index, we aim to determine the direction of these differences using a single-task contrast. The results indicated that the group with the highest mean Global WL Index was the team sports group, with Welch’s test revealing a statistically significant difference (T = –2.16, df = 54.6, p = .018). This suggests that, on average, the individual sports group has a lower Global WL Index than the team sports group.

Differences were also observed in somatic anxiety between team and individual athletes. When testing the one-tailed (unilateral) hypothesis, results indicate that the individual sports group exhibited higher levels of somatic anxiety than the team sports group. A Mann-Whitney test confirmed a statistically significant difference in Somatic Anxiety (U = 933, p = .012), supporting this finding. However, no significant differences were found for Worry (U = 1063, p = .101) or Deconcentration (U = 1177, p = .346).

### Clustering

**Figure 1:**
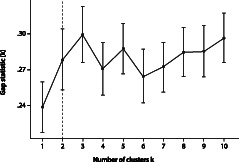
Optimal number of clusters suggested by K-Means Clustering technique

A clustering analysis was conducted using the K-means technique. The optimal number of clusters was determined to be two *(Figure 1)*. The objective of this analysis was to group the psychological variables evaluated as psychological profiles.

Examining both the centroid table *(Table 8)* and the dendrogram, two distinct psychological profiles emerged, particularly regarding Frustration and Performance.

**Table 8:** Centroids of cluster factors

	Frustration Weight	Effort Weight	Worry Mean	Somatic Mean	Physical Weight	Performance Weight
1	0.762	3.536	2.202	2.155	3.464	3.476
2	4.174	2.609	2.891	2.761	2.043	1.696

*The first profile* was characterized by low frustration, high effort, lower anxiety levels, higher physical demand, and high performance.

*The second profile* showed high frustration, low effort, medium-high levels of anxiety, and low physical demand and performance.

**Figure 2:**
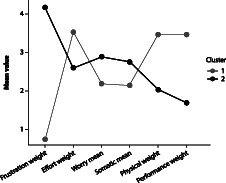
Plot of means across Clusters (K-Means Clustering)

These profiles are more clearly visualized in the Plot of Means illustration *(Figure 2).*

## Discussion

The results of this study indicate that the perception of mental workload (WL) is multifactorial and should be analyzed on various levels, extending beyond purely descriptive aspects related to the practice of the studied sports.

The high level of competition intensity and training experienced by the sample participants lends significant relevance to the results and their interpretation, providing valuable insights into the psychological mechanisms underlying their predisposition to sports performance.

The initial data analysis suggests that certain sports, such as canoe-kayak, wrestling, skating, and handball, report higher physical demands than others. These same sports also demonstrate higher overall workload values as measured by the Global WL Index.

Examining the list of sports studied, it is notable that these differences emerge, especially when compared to sports that might initially seem more demanding. The sports with the lowest perceived physical demands are sambo and track and field athletics.

Wrestling stands-out as particularly significant in terms of workload (WL). As will be discussed later (Rogaleva et al., 2024), this may be attributed to group and social pressure to excel in wrestling due to the cultural significance of this sport and its emphasis on achieving high performance.

One factor that underscores the importance of considering other factors, such as decision-making, automation of sports movements, and associated injury risks (Gao et al., 2024), is highlighted by the observation that skiing and snowboarding have the highest mental demands, while dance is perceived as having the most significant time pressure, despite not being the only sport with a unique time-dependent execution requirements (Pittman et al., 2005).

When examining the relationship between these values and anxiety associated with competition and performance, the results are striking, even at first glance. It appears that perceived effort, the amount of energy expended to perform an athletic action, may serve as a protective factor against competitive anxiety, as these two variables consistently exhibit a negative correlation. This interpretation gains reliability when considered alongside more typical findings (Núñez & Garcia-Mas, 2017), such as the relationship between performance-related anxiety and frustration or between somatic anxiety and time pressure. Therefore, observing the overall results, it appears that as effort increases in one direction, frustration (across both WL factors) decreases.

As the relationship between burnout and workload (WL) is further analyzed, intriguing data emerges that complement the previous findings. Specifically, as overall perceived WL increases, reflecting greater physical and mental demands, levels of performance-related anxiety and issues of concentration tend to decrease among athletes. These findings suggest that WL may act as a protective factor. Somatic anxiety appears at very low levels, almost exclusively related to time demands in certain sports. On the other hand, a high Global WL Index correlates predictably with the Exhaustion factor, which has a strong physical component. In a similar positive correlation, increased WL appears to alleviate the pressure on athletes to demonstrate higher levels of personal commitment, as they already perceive themselves as performing well, both physically and mentally.

These findings raise important questions for future research, particularly regarding their implications for training programs and competition structures. Moreover, they highlight the counterintuitive nature of psychological dynamics in high-performance sports.

Building upon these findings and prior to an exploration of potential sporting group classifications, it should be noted that eudaimonic psychological well-being shows no significant positive or negative relationship with any of the research variables. This finding contrast starkly with similar studies (Carson, et al. 2020). This may be due to cultural bias that influences the understanding of eudaimonic well-being, suggesting that perhaps hedonic well-being is more deeply ingrained in the mindset and cognitive framework of the athletes studied. This is a crucial area warranting further investigation, potentially even from an anthropological perspective.

The findings of this research align with existing literature on individual and team sports. Higher levels of anxiety, particularly somatic anxiety, has been observed most significantly among athletes competing in individual sports. In some cases, this can be attributed to the fact that responsibility rests solely on the athlete, whereas in team sports, responsibility is distributed among team members, reducing the psychological burden (Bandura, 1991; Corrion et al., 2009). In contrast, higher levels of WL, as indicated by the Global WL Index, are found in team sports. This finding supports the premise underlying the workload framework proposed by Cardenas et al. (2015), as it demonstrates that WL is a product of systemic entropy. In the context of sports, a system can be defined by the interaction between two components: a) the specific sport and its characteristics, and b) the athletes themselves, with their learning history, expectations, and beliefs. The greater the uncertainty (ie. entropy) within a task, the more resources it demands; leading to higher emotional strain and, consequently, higher perceived WL by the athletes. These observations align with our research results, as the entropy of a sport or task, according to Cardenas et al., 2015 are determined by four cognitive parameters: numerical relationships of players, degrees of freedom, available time, and available space. Considering these parameters, it becomes evident that team sports, as more *entropic* systems, exhibit higher WL values in the Global WL Index.

The variance in anxiety levels across groups have traditionally been attributed to the concept of shared responsibility. However, based on these findings, we can hypothesize that the higher levels of WL may also influence this difference assuming WL functions as a protective factor.

Finally, the clustering analysis identified two distinct athlete profiles based on the previously analyzed factors:

1. The *bright side* profile that includes athletes who perceive high levels of effort and physical demand, while experiencing low levels of anxiety and frustration.
2. The *dark side* profile that includes athletes who exhibit high levels of anxiety and very high frustration, but report low perceived effort and physical demands.

## Conclusion

This study yields three key conclusions. First, workload (WL) in athletes is multifaceted, influenced by both physical and mental factors, and may serve as a protective factor against anxiety. Due to their higher *entropy* (environmental uncertainty), team sports exhibit higher WL levels than individual sports. This increased WL may help reduce performance-related anxiety and enhance concentration. Second, individual sports athletes experience higher levels of somatic anxiety, likely due to greater personal responsibility. In contrast, team sport athletes report lower anxiety levels but higher WL levels, reflecting the demands and complexity of team interactions. Finally, two contrasting athlete profiles emerge including bright side and dark side profiles These profiles highlight how athletes’ perceptions of workload and demand-types shape their emotional experiences in sports.

Future research should explore workload management interventions aimed at optimizing the balance between perceived effort and competitive anxiety through tailored approaches. Additionally, a deeper understanding of the *bright* and *dark* athlete profiles as this may provide valuable insights for the development of psychological training programs, particularly with respect to efforts to reduce frustration levels among athletes within the dark profile segment.

Additionally, the examination of the role of *entropy* in team versus individual sports could offer new strategies for improving psychological preparation.

From a cultural perspective, continued investigation into factors affecting eudaimonic well-being is essential. Finally, studying the relationship between mental workload (MW) and burnout may provide valuable insights into reducing burnout through more effective training load management in high-performance athletes.

## Limitations

While this exploratory study concerning WL in sports provides suggested future areas of WL research including its theoretical and practical implications, it leaves open the question of assessment tools. Anxiety, burnout, and well-being were evaluated using selected items from three validated instruments in order to explore their relationships. Additionally, while the NASA-TLX is one of the most widely used questionnaires for assessing WL across various domains, it is not specifically designed for athletes.
